# Aspergillose pulmonaire chronique nécrosante à *Aspergillus niger* chez un patient tabagique et ancien tuberculeux

**DOI:** 10.11604/pamj.2014.17.93.3631

**Published:** 2014-02-06

**Authors:** Ghita Yahyaoui, Imane Tlamçani, Salma Benjelloun, Mohamed Atwani, Mohamed Errami

**Affiliations:** 1 Service de parasitologie, hôpital militaire Moulay Ismail de Meknès, Maroc; 2 Service de chirurgie thoracique, hôpital militaire Moulay Ismail de Meknès, Maroc

**Keywords:** Aspergillose pulmonaire chronique nécrosante, Aspergillus niger, diagnostic, voriconazole, Chronic necrotizing pulmonary aspergillosis, Aspergillus niger, diagnosis, voriconazole

## Abstract

Nous rapportons le cas d'une aspergillose pulmonaire chronique nécrosante chez un patient tabagique et ancien tuberculeux. Le diagnostic a été basé sur des critères radiologiques, tomodensitométriques et mycologiques. Le champignon a été isolé des crachats et de la pièce d'exérèse. En plus du traitement chirurgical, un traitement médical à base de voriconazole a été instauré. Une dose de charge de 600mg a été administrée le premier jour sous forme de deux injections intraveineuses espacées de 12 heurs, ensuite 400mg par jour répartie en deux prises matin et soir. Après 45 jours de traitement, une amélioration clinique et radiologique a été déjà observée. Lors d'aspergillose pulmonaire chronique nécrosante, un traitement antifongique de longue durée parait être nécessaire. Le Maroc est un pays bien ensoleillé, notre malade risquerait de développer une photosensibilisation. En plus l'itraconazole pouvant être une bonne alternative thérapeutique n'est pas disponible sur le marché national.

## Introduction

L'aspergillose pulmonaire chronique nécrosante (APCN) ou aspergillose pulmonaire semi-invasive est une forme clinique rare d'infection aspergillaire. Elle a été individualisée pour la première fois par Gefter et al et Binder et al en 1981 [1].

Il s'agit d'une invasion locale indolente du parenchyme pulmonaire par un champignon du genre *Aspergillus* aboutissant à une destruction tissulaire avec ou sans cavitation [2, 3]. Cette entité clinique est différente de l'aspergillome qui correspond à une colonisation par le champignon d'une cavité préexistante aboutissant à la formation d'une boule fongique sans extension locale [4]. L'APCN est également différente de l'aspergillose pulmonaire invasive par son évolution plus lente qui se fait sur plusieurs mois voire plusieurs années sans invasion vasculaire ni dissémination vers d'autres organes [2].

*Aspergillus niger* est en général reconnu comme étant une espèce peu invasive impliquée dans la colonisation des conduits auditifs ou des sinus [5]. Ses spores sont relativement plus grandes que celle *d'Aspergillus fumigatus*. Elles sont habituellement captées par le système mucociliaire de l'hôte et seraient moins responsables d'infections de l'arbre respiratoire inférieur comparativement à l'espèce *A. fumigatus* [6]. Nous rapportons le cas d'une APCN à A. niger chez un patient tabagique ayant un antécédent de tuberculose pulmonaire.

## Patient et observation

**Figure 1:**
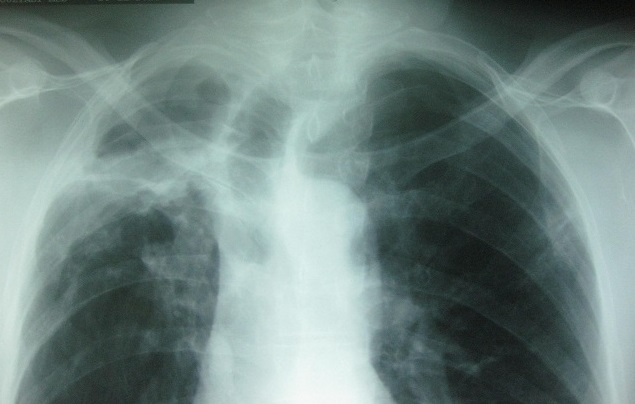
Radiographie pulmonaire montrant une opacité au niveau du lobe supérieur droit avec infiltrats parenchymateux adjacents

**Figure 2:**
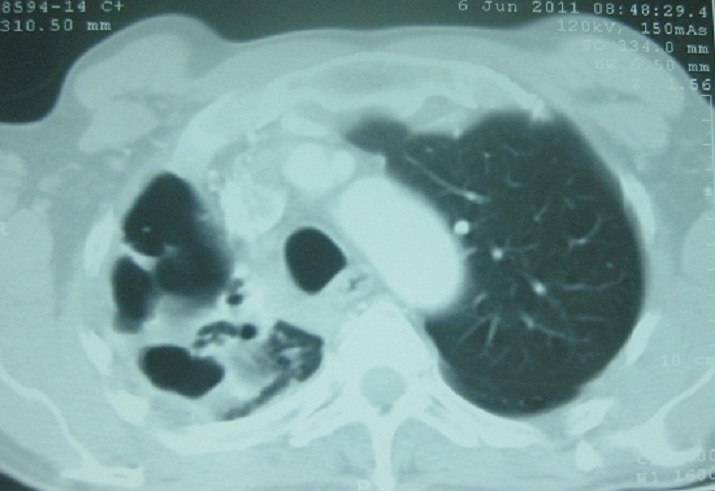
TDM thoracique montrant la présence d'image cavitaire au sein d'une condensation parenchymateuse pulmonaire apicale droite au contact avec une bronche segmentaire en regard

Il s'agit d'un patient de sexe masculin, âgé de 48 ans, tabagique chronique, fumant la cigarette avec filtre et rapportant ne pas avoir fumé le cannabis pendant les deux dernières années. Il avait comme antécédent une tuberculose pulmonaire traitée il y a deux ans. Il a été hospitalisé au service de pneumologie pour hémoptysies modérées. A l'admission, il était en assez bon état général et apyrétique. La recherche de BK sur les crachats était négative. L'hémogramme montrait un taux d'hémoglobine à 12 g/dl, les leucocytes à 6500/mm^3^ avec des polynucléaires neutrophiles à 4500/mm^3^. Le bilan inflammatoire comprenait une VS (1^ére^ heure) à 95mm et une CRP à 18mg/l. Le reste du bilan biologique était sans particularité. La radiographie pulmonaire révélait la présence d'une opacité apicale droite avec infiltrats parenchymateux adjacents (Figure 1). La tomodensitométrie thoracique montrait la présence de deux images cavitaires au sein d'une condensation parenchymateuse pulmonaire apicale droite au contact avec une bronche segmentaire en regard avec dilatation des bronches (Figure 2). Cet aspect était évocateur d'une infection aspergillaire. Une sérologie recherchant les anticorps anti-*Aspergillus fumigatus* a été alors demandée et revenait négative. Un prélèvement des crachats pour examen mycologique nous a été adressé.

**Figure 3:**
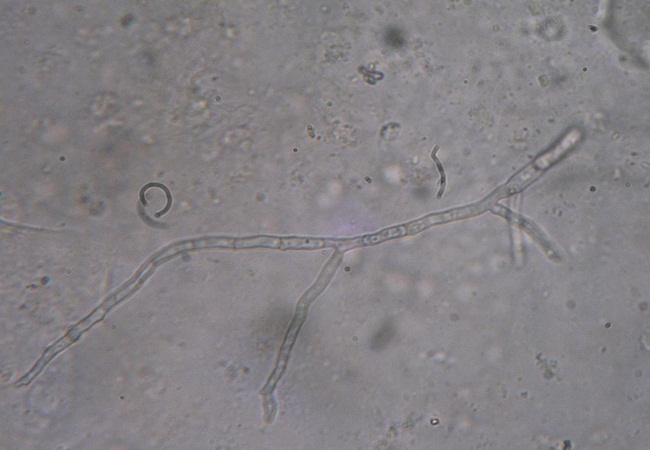
Examen direct entre lame et lamelle (grossissement x 400) d'un prélèvement de culot de centrifugation du crachat montrant un filament mycélien cloisonné et ramifié

L'examen direct entre lame et lamelle d'un prélèvement du culot de centrifugation a révélé la présence de filaments mycéliens septés et ramifiés (Figure 3). La culture sur milieu Sabouraud-choramphénicol a été réalisée et incubée à 28°;C. Des colonies qui étaient d'abord blanches et devenant de plus en plus granuleuses et noires ont poussé sur le milieu. L'examen entre lame et lamelle d'un prélèvement de ces colonies a mis en évidence la présence de têtes aspergillaires bisériées recouvertes entièrement par des spores globuleuses, brunâtres et rugueuses. Le champignon a été alors identifié comme étant *A. niger*. Une sérologie pour la recherche d'anticorps anti-*Aspergillus niger* a été par la suite demandée et revenait positive.

**Figure 4:**
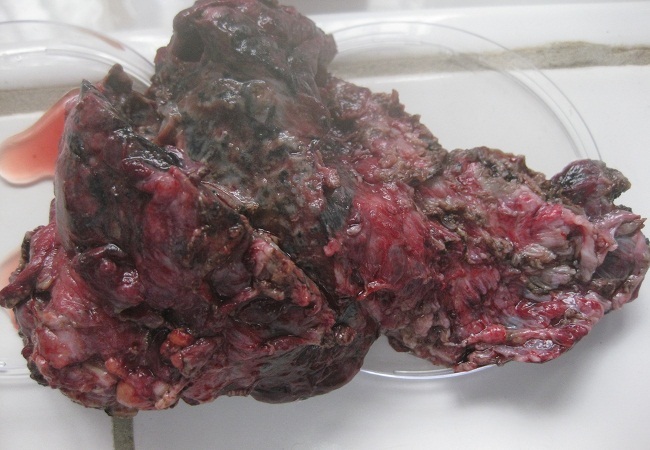
Pièce de lobectomie après exérèse chirurgicale montrant une destruction importante du parenchyme pulmonaire par le champignon

**Figure 5:**
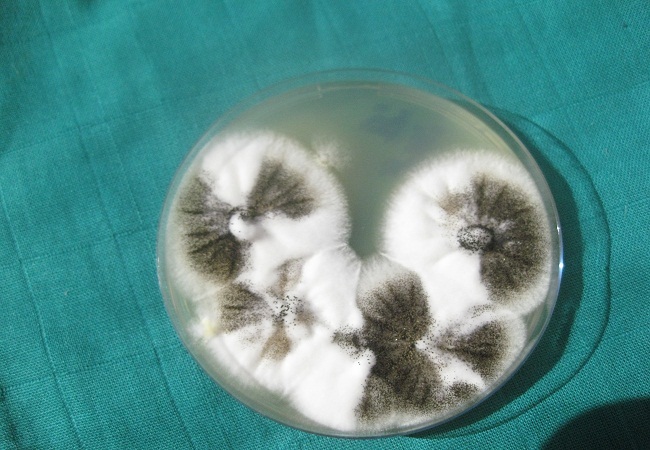
Culture d'Aspergillus niger sur milieu Sabouraud-chloramphénicol montrant des colonies jeunes blanches avec des grains noirs à leur surface

Une lobectomie supérieure droite a été réalisée. L'examen anatomo-pathogique de la pièce opératoire a montré la présence de 2 excavations aspergillaires avec réaction inflammatoire granulomateuse au contact et présence filaments mycéliens et de corps étrangers biréfringents à l'examen en lumière polarisée. L'examen direct et la culture mycologique d'un prélèvement de cette pièce étaient positifs et permettaient d'isoler à nouveau *A. niger* (Figure 4, Figure 5).

**Figure 6:**
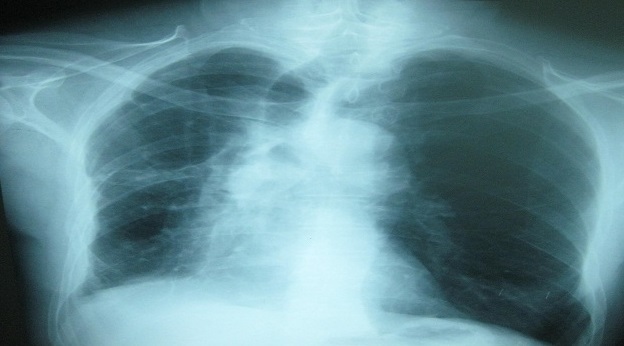
Radiographie pulmonaire réalisée après 45 jours de traitement montrant une amélioration radiologique (disparition des infiltrats)

Le malade a été traité par le voriconazole (Vfend^®^) en une dose de charge de 600mg/jour répartie en deux injections IV espacées de 12 heures le premier jour ensuite 400mg/j répartie en deux prises matin et soir. Ce traitement était administré par voie IV pendant 10 jours, ensuite par voie orale. Au bout de 42 jours de traitement, une amélioration clinique et radiologique a été déjà observée (Figure 6).

## Discussion

L'APCN survient chez les sujets d’âge moyen présentant une immunodépression modérée en rapport avec un diabète, un alcoolisme, une hépatopathie chronique, une corticothérapie à faible dose, une malnutrition ou une connectivite. Elle survient également chez les patients ayant une altération des défenses locales notamment ceux présentant une broncho-pneumopathie chronique obstructive ou une fibrose pulmonaire ainsi que ceux ayant des séquelles de tuberculose pulmonaire, de chirurgie thoracique ou de sarcoïdose [1,24,7]. L'association d'un tabagisme chronique, par ses effets néfastes sur les tissus pulmonaires, serait favorable au risque d'infection pulmonaire. Le tabac, au même titre que le cannabis sont souvent contaminés par des spores fongiques [8]. Le cannabis, fumé habituellement sans filtre ou manipulé, a été associé avec diverses formes d'infections aspergillaires [9]. La cigarette sans filtre serait également une source de spores aspergillaires. Notre patient a rapporté l'utilisation de tabac avec filtre. Néanmoins, l'association d'un tabagisme chronique aux séquelles tuberculeuses accentuerait le risque de développement d'infection aspergillaire. L'arrêt du tabac serait par conséquent une mesure favorable au succès thérapeutique.

Sur le plan clinique, en plus d'une toux productive et d'une hémoptysie. L'APCN se manifeste par des signes généraux tels qu'une fièvre, asthénie et perte progressive de poids. Occasionnellement elle pourrait être asymptomatique [1].

Sur le plan radiologique, l'imagerie par radiographie montre habituellement des infiltrats avec ou sans cavitations essentiellement au niveau des lobes supérieurs. Dans environ 50% des cas un aspergillome est associé [2,24].

L'imagerie par TDM montre souvent des images de consolidation et d’épaississement pleural [1]. Le diagnostic sérologique se fait par la recherche d'anticorps précipitants par des techniques d'immunodiffusion telles que l'immunoélectrophorèse ou l’électro synérèse. Il se fait également par la recherche de divers anticorps par des techniques d'immunomarquage: ELISA, HAI et IFI [10]. Dans l’écrasante majorité des cas, la sérologie est positive avec présence des IgG [1,24]. Dans de rares cas, elle pourrait être négative [4]. La réaction cutanée immédiate aux antigènes aspergillaires pourrait être une aide au diagnostic [1, 2]. Les marqueurs de l'inflammation notamment la VS et la CRP sont habituellement élevés [4]. La mise en évidence du champignon se fait sur le liquide de lavage broncho-alvéolaire ou sur des expectorations. Les produits d'expectorations présentent l'inconvénient de ne pas être profonds avec un risque de résultats faussement négatifs ou d’être parfois contaminés par des Aspergillus pouvant être de simples colonisateurs des voies aériennes supérieures à la faveur de résultats faussement positifs.

L'examen direct se fait par montage du culot de centrifugation du prélèvement entre lame et lamelle et observation au microscope d'abord au grossissement x 100 ensuite x 400. Il permet de mettre en évidence des filaments mycéliens septés et ramifiés. La culture se fait par ensemencement du reste du culot de centrifugation sur milieu Sabouraud-chloramphénicol et incubation à 28°C.

Le délai de pousse des *Aspergillus* est de 2 à 4 jours. L'identification d'espèce repose sur l'aspect macroscopique des colonies et l'aspect microscopique des têtes aspergillaires observées sur le prélèvement de ces colonies. A. niger est une espèce facile à identifier d'après seulement l'aspect macroscopique des colonies: lorsque ces dernières sont jeunes, elles ont une couleur blanchâtre avec des grains noirs (têtes aspergillaires) à la surface. L'examen histopathologique se fait le plus souvent sur pièce de lobectomie. Il met en évidence une inflammation de la paroi cavitaire avec nécrose tissulaire associée à la présence de filaments mycéliens *d'Aspergillus*. Dans le cas d'infection par *A. niger*, il y a souvent présence de cristaux d'oxalate de calcium [6, 7, 11]. Ils résultent d'une précipitation au pH physiologique de l'acide oxalique (mycotoxine) secrété par *A. niger* après réaction avec le calcium sanguin et tissulaire [7, 11]. D'autres espèces *d'Aspergillus*, notamment *A. flavus* et *A. fumigatus* peuvent plus rarement secréter cette substance [7]. Pour notre patient, les cristaux biréfringents observés à la lumière polarisée lors de l'examen histopathologique seraient vraisemblablement ceux d'oxalate de calcium.

L'amphotéricine B à la dose de 0.5 à 1mg/kg/j pour la forme desoxycholate et de 3 à 5mg/kg/j pour les formulations lipidiques a été utilisée avec une bonne efficacité thérapeutique. L'itraconazole à la dose de 400mg/j a été par la suite une bonne alternative à la toxicité de l'Amphotéricine B. Récemment le voriconazole a émergé comme étant le traitement de première ligne. Il s'est montré plus efficace lors d'APCN que lors des autres formes d'aspergillose pulmonaire chronique [12].

Il est administré en une dose de charge de 600mg répartie en deux injections IV le premier jour puis 400mg/j réparties en deux prises matin et soir. Ce traitement se fait par voie intraveineuse pendant 7 à 10 jours, ensuite par voie orale. La durée idéale du traitement n'est pas encore bien définie, elle dépendrait du degré d'extension du processus infectieux, de la réponse au traitement, de facteurs pulmonaires locaux et de l’état immunitaire du patient [12]. Parfois, un traitement à vie pourrait être nécessaire [2–4].

Une différence interindividuelle a été observée dans le métabolisme du voriconazole à l'origine d'une grande variabilité des taux sériques de ce médicament. Un suivi thérapeutique par le dosage du taux de la molécule dans le sang serait une précaution importante surtout en cas de mauvaise réponse au traitement [3, 6].

En plus le voriconazole, quoique moins toxique que l'amphotéricine B, peut avoir certains effets indésirables gênants voire graves. Les plus fréquents sont des troubles visuels: vision non claire, photophobies, altération de la perception des couleurs. Moins fréquemment, on peut observer des troubles hépatiques ou cutanés en particulier une photo toxicité ou même photo carcinogenèse si le traitement est maintenu de manière prolongée [1, 13]. Des mesures de photo protection devraient alors être entreprises en cas de prescription de voriconazole [13] et le relais par l'itraconazole pourrait être choisit en cas de survenue de tels effets indésirables. Le Maroc est un pays bien ensoleillé, notre malade risquerait de développer une photosensibilisation. En plus l'itraconazole pouvant être une bonne alternative thérapeutique n'est pas disponible sur le marché national.

Lors d'APCN, un traitement chirurgical mérite d’être associé vue la faible pénétration des antimycosiques dans les tissus infectés [14]. Ce traitement permettrait aussi de diminuer l'inoculum améliorant ainsi l'efficacité des antifongiques [5]. La lobectomie serait l'intervention de référence [14]. Elle reste toutefois réservée aux patients dont l’état général et pulmonaire le permet [1].

L’évaluation de la réponse au traitement se fait par un suivi clinique, radiologique, sérologique et mycologique [4]. Le pronostic à long terme de l'APCN n'est pas bien documenté [2], Il dépendrait de la nature de la prise en charge thérapeutique, de comorbidité pulmonaire associée ainsi que de l’état immunitaire du patient [15]. Des cas, dont l'infection était due à *A. niger* ayant évolué vers une dissémination fongique ont été décrits [5, 16]. La mortalité liée à cette pathologie a été estimée entre 10 et 40% [2, 15].

## Conclusion

L'aspergillose pulmonaire chronique nécrosante reste une forme rare de l'atteinte pulmonaire, et se distingue des autres infections aspergillaires respiratoires par sa forme clinique et son évolution particulières. Seulement quelques cas sont rapportés dans la littérature. Malgré le caractère exceptionnel de cette forme d'aspergillose et la difficulté d'envisager des recommandations étayées par les résultats de grandes séries, nous pensons que la chirurgie pourrait être proposée initialement en association avec le traitement médical dans la prise en charge des APCN. L'efficacité attendue de médicaments récents (voriconazole, amphotéricine B, itraconazole) peut envisager d'espérer une amélioration du pronostic de ces patients.
